# Dynamics of visual reversals from ambiguous spinning biological-motion and rigid structure-from-motion

**DOI:** 10.1177/20416695251342410

**Published:** 2025-05-21

**Authors:** Leo Poom

**Affiliations:** 1 8097 Uppsala University, Sweden

**Keywords:** biological motion, structure-from-motion, bi-stable, ambiguous, perceptual reversals

## Abstract

Three-dimensional rigid structure-from-motion (SFM) and structure from nonrigid biological point-light motion stimuli are perceptually ambiguous. This study investigated the dynamics of perceived reversals in two cases: a spinning point-light walker (PLW) and a spinning rigid human figure in a walking pose (SFM). It specifically focused on two key questions: Could the facing-the-viewer bias (FTV) account for the reversals for spinning PLW? To what extent do motion cues from limb motions or configural cues from the human shape contribute to the perceived reversals? In Experiment 1, participants reported reversals with more than twice the frequency while viewing the upright and inverted PLW than for the rigid structures, but an FTV bias was observed only for the upright walker. The perception of an ambulating living human shape of typically encountered walkers in an upright position thus plays a crucial role in obtaining an FTV bias for these spinning stimuli. In Experiment 2, the human figures walked or rigidly moved along a circular path while facing the motion direction, spinning at the same rate as in Experiment 1. A strong initial FTV bias was then observed, but the reversal rate was substantially reduced compared to reversals when spinning on the same spot. These findings highlight theoretically interesting distinct temporal dynamics of reversals and biases between biological motion and rigid SFM. It is argued that the differences in reversals between conditions have a common cause in the form of past experiences that differ between conditions.

## How to cite this article

Poom, L. (2025). Dynamics of visual reversals from ambiguous spinning biological-motion and rigid structure-from-motion. *i-Perception, 16(0)*, 1–22. https://doi.org/10.1177/20416695251342410

## Introduction

In visual perception research, a common strategy is to minimize the information in stimuli to study the visual system's isolated use of specific cues. Using this strategy, Wallach and O’Connell (1953) demonstrated that the visual system could quickly recover rigid 3D structures from the 2D motion of shadows cast by rotating 3D wire-frame figures. About 50 years ago, Johansson (1973) showed that human observers could effortlessly recover biological structures and actions from point-light displays where dots represent the main joints of a human in motion, involving bending at the joints. He coined the term “biological motion” for these displays. Though “biological motion” can refer to various types of animate movements (Troje, 2013), here I use it as originally defined by Johansson (1973). Another influential motion stimulus paradigm is rigid structure-from-motion (SFM) (Ullman, 1979). Rigid SFM refers to perceiving the 3D shape of an inanimate object solely from the relative movements of dots across multiple 2D images, for example, produced by the spinning motion of a rigid object, although only a random collection of dots is visible in a single image. A similarly spinning biological motion in the form of a point light walker (PLW) can easily be identified although the motion is much more complex since it is the sum of the common rotation and the periodic motions of the limbs. Prior knowledge of the structure, especially when the spinning PLW is inverted, facilitates the decomposition of the overall motion and the periodic motions of the limbs (Pastukhov, 2017). Spinning SFM and PLW stimuli are ambiguous, and the perceived spinning direction reverses back and forth during observation. A spinning PLW reverses with a much higher reversal rate than a spinning rigid SFM cylinder (Jackson et al., 2008). Also, biases appear for some ambiguous stimuli: A PLW typically appears as facing the viewer (FTV) rather than facing away (Vanrie et al., 2004), but it is not known if this bias is caused by the recognizable human figure or if the limb motions of the human figure are crucial for the high reversal rates and the FTV-bias.

Some theories propose shared processes for recovering nonliving rigid SFM and jointed biological motion (e.g., Johansson, 1973; Wallach & O’Connell, 1981), while other models suggest specialized processes for biological motion (e.g., Hoffman & Flinchbaugh, 1982; Giese & Poggio, 2003; Troje & Chang, 2023). Computational models indicate that biological motion identification can be achieved without local motion by using whole-body posture templates that integrate information over time (Lange & Lappe, 2006). A template model could be used to recover rigid SFM as well. The fact that humans can recover shape from limited dot-lifetime biological motion displays, where dot positions shuffled between frames are temporally integrated, supports such models (Beintema & Lappe, 2002). Other studies have shown that human biological motion perception requires intact motion processing (Garcia & Grossman, 2008). In addition, distinct processes may be used to detect biological motion via bottom-up signals conveyed by local motion of individual dots that activate specialized detectors, and identification of the global organization from the relative motions of the dots (Troje & Chang, 2023). Different bottom-up mechanisms and/or the involvement of diverse top-down processes (Kroustallis, 2004; Pastukhov, 2017) may account for the empirical evidence suggesting different processes involved in perceiving rigid SFM and biological motion (Neri et al., 1998; Poom & Olsson, 2002; Shiffrar et al., 1997). Thus, the ability to detect and identify global shape from biological motion may share processes used to recover nonliving structures but involve additional processes not required for detecting nonliving SFM.

**Figure 1.**
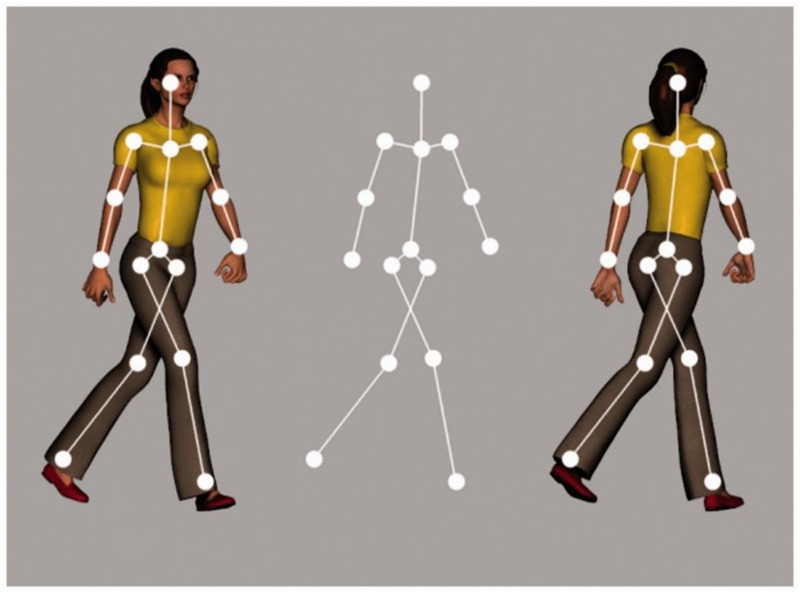
The Point-Light Walker, shown here as a stick-figure, is ambiguous and can be perceived as facing toward or away from the observer. Observers are subject to a facing-the-viewer bias where the left interpretation is perceived rather than the right (from Figure 1 in Weech & Troje, 2018, distributed under the terms of the Creative Commons Attribution 4.0 License).

Past interactions with other humans likely shape the top-down processes involved in biological motion perception (Kroustallis, 2004). Johansson (1973) acknowledged the role of top-down influences from past experiences since he observed that inverted biological motion stimuli are much harder to recognize than upright stimuli. Likewise, prolonged exposure to microgravity during spaceflight, and microgravity using a head-down-tilted bed rest, reduces the inversion effect by Wang et al. (2022). Such top-down processes can be probed using ambiguous stimuli. Parallel projections of rigid SFM and biological motion, devoid of perspective and occlusion cues, do not distinguish between the front and back views and are perceptually ambiguous. A point-light walker (PLW) is ambiguous regarding its facing direction (Figure 1). Consequently, the perceived depth order of ambiguous SFM stimuli typically reverses during prolonged viewing, which can be used to investigate the influences of endogenous processes, such as repeated adaptation-recovery cycles among neural populations (Nawrot & Blake, 1989), and contributions of top-down influences (Megumi et al., 2015; Poom, 2024; Poom & Matin, 2022; Weilnhammer et al., 2017). Differences in past experiences are known to influence the perception of ambiguous stimuli. Examples are the view-from-above bias, resulting from our experience with a ground level and gravity, that disambiguates wire-frame figures like the Necker cube (Clément et al., 2015), and also influences the perception of ambiguous biological motion (Zhang et al., 2017).

An ambiguous PLW displayed as walking on a treadmill is preferably perceived as facing the viewer rather than facing away (Vanrie et al., 2004). This FTV bias is similar to the hollow face illusion, where a face mask is seen as bulging outward regardless of whether it is viewed from the front or back. The hollow face illusion is likely influenced by prior experiences of faces (Gregory, 1970) as well as the convexity bias (Corrow et al., 2014; Hill & Bruce, 1994) where surfaces with ambiguous bulging are perceived as bulging outward (Liu & Todd, 2004), due to the higher occurrence of surfaces and objects bulging outward than inward. The FTV bias likely stems from multiple causes. The convexity bias contributes to the FTV bias since observers have a preference to perceive the knees as bulging toward rather than away from the observer, and their attentional preference is directed to the lower body parts (Schouten et al., 2011; Weech et al., 2014). Accordingly, when presenting only the upper body of a PLW it tends to be perceived as facing away (Schouten et al., 2011), and an inverted nonspinning PLW results in a facing-away bias in line with the convexity bias since the elbows are the lower part of an inverted walker (Weech & Troje, 2018). The FTV bias may also involve a strategy to avoid costly mistakes in perceiving an approaching person as receding (Heenan & Troje, 2014; Vanrie et al., 2004). Studies have shown that social anxiety and the FTV bias may be related, which supports a social interpretation of a PLW, but the relation is inconsistent (Heenan & Troje, 2014; Peng et al., 2021; Van de Cruys et al., 2013). Sex differences (Schouten et al., 2010) and factors like physical exhaustion from exercise also influence the FTV bias (Heenan & Troje, 2014).

An additional cause to the FTV bias may arise as a consequence of visual attention being attracted toward important and interesting stimuli. Other people approaching are more important to keep track of than receding people and therefore likely to attract attention to a higher degree. This preference for approaching rather than receding people may also contribute to the FTV bias. Although, to my knowledge, there is no direct evidence from eye tracking studies for an attentional preference toward other people facing the observer, there is indirect evidence from brain imaging studies. It is known that increased cortical activity indicates heightened attention (Moran & Desimone, 1985) and that biological motion elicits activity in the superior temporal sulcus (STS) in the brain (Grossman et al., 2000). Activity in STS is greater for walkers gazing toward the observer than gazing away (Pelphrey et al., 2004), suggesting heightened attention. The biological motion also elicits activity in frontal regions involving motor systems (Saygin et al., 2004), and observed humans involved in activities facing the observer elicit stronger motor system responses than those facing away (Kilner et al., 2006). Thus, an FTV bias could result from this attentional preference toward other people walking and facing toward the observer rather than walking and facing away.

A PLW displayed as walking on a treadmill rarely reverses and is preferably perceived with an FTV bias. A spinning PLW, on the other hand, reverses with a much higher reversal rate than a spinning rigid SFM cylinder (Jackson et al., 2008). Although identification of a spinning PLW requires successful disentanglement of the coherent spinning from the periodic pendulum motions of the limbs, observers in the Jackson et al. study could effortlessly accomplish this. Furthermore, reversals of the whole configuration of a human shape occur in concert, different parts do not reverse independently or in asynchrony, suggesting a global process. In addition, an FTV bias has been reported for a spinning walking stick-figure, and a rigidly spinning human stick-figure in a walking posture, but not a spinning standing human stick-figure (Weech et al., 2014). This suggests that it is the global shape of the stick-figure in a walking posture (whether walking or not) that triggers the high reversal rate. It is uncertain, however, whether the results from Weech et al. (2014), using a spinning stick-figure whose shape can be recovered from static frames, can be replicated with a spinning PLW where the global configuration has to be completed between the dots (Giese & Poggio, 2003; Troje & Westhoff, 2006), possibly aided by top-down processes (Pastukhov, 2017). Whereas the identification of a spinning PLW likely requires globally distributed attention, the explicit shape of a stick-figure could direct attention to the lower body parts to result in a convexity bias at the knees. Similarly, a rigidly spinning human point light figure in a walking pose whose parts are only involved in a common rotation could also attract attention to the lower body parts which could result in a convexity bias causing the overall FTV bias. On the other hand, a rigid human shape poses no threat or expected social interaction, which could reduce the FTV bias according to the hypothesis that the FTV bias involves social interpretations of the stimuli (Vanrie et al., 2004). Additionally, past occurrences of perceived other people walking in circles are likely more frequent than occurrences of walking people spinning in the same place. A spinning PLW could thus trigger the more likely alternative interpretation that the figure reverses in a spinning direction to maintain facing the viewer rather than spinning in the same direction. Thus, multiple factors, such as the convexity bias, cognitive strategies, and attentional bias from past occurrences, may differently influence the reversal rate and the facing-the-viewer bias of a spinning PLW and SFM of a rigidly spinning human in a walking pose.

Several questions are addressed here: Are the rapid reversals found for a spinning PLW (Jackson et al., 2008), and the FTV bias (Vanrie et al., 2004), produced by the limb motions, or are the rapid reversals and FTV bias caused by the human shape, and are reversals influenced by the effortless identification of an upright figure? Also, the rapid reversals for a spinning PLW could be due to the rare prior experiences of observing spinning walkers, so alternative interpretations are preferred. The prior occurrences of observing other people turn while moving along the ground by walking are likely much more frequently observed than walkers spinning at the same spot. To address this question, in Experiment 2, a PLW and a rigid human figure in a walking posture (SFM) were turning as a result of moving along a circular path while reversals in the direction along the path were counted.

The aim of Experiment 1 was to compare the circular distributions of reversals, average reversal rates, and FTV biases for a spinning human point-light walker (PLW), a rigidly spinning human shape (SFM). Additionally, an inverted spinning PLW, an inverted rigidly spinning human shape, and a scrambled rigidly spinning structure were included to explore influences of prior experiences of upright figures and the influence of a recognizable human shape on the reversals and the FTV-bias. Experiment 1 addressed the following questions: Is the rapid perceptual reversals of a spinning PLW (as previously reported by Jackson et al., 2008) caused by the human shape in a walking pose, and does the shape of a human figure in a walking pose result in an FTV bias? The inverted spinning walking and rigid stimuli were used to investigate whether perceptual experiences of upright biological motion influence reversals and attempt to replicate the reverse FTV bias for an inverted nonspinning PLW (Weech & Troje, 2018). The scrambled rigid shape was included to investigate whether stimuli in the form of a human shape had any influence on reversals. Finally, correlations between reversal rates in different conditions were calculated to explore possible common processes involved in causing reversals for upright and inverted walking and rigid figures. If common processes are involved to a high degree, similar circular distributions of reversals, reversal rates, and FTV biases were expected, and reversal rates should be strongly correlated.

The aim of Experiment 2 was to explore reversal rates for a rigid and a walking human moving/walking in circles in upright conditions while facing the motion direction. The human figures were spinning at the same rate as in Experiment 1 (360ͦ° for every completed turn around the circle). Observers likely have more perceptual experiences of other people walking in circular paths, or at least turning while moving forward, than spinning in place. Since spinning walkers are rarely experienced in natural conditions, other more likely interpretations (with a high prior), such as reversing the spinning direction to maintain an FTV interpretation could outweigh the continuous spinning interpretation of the sensory information. Thus, in this view, if the spinning of the PLW can be explained by walking in a circular path, then reversals may seem less likely than if the PLW is spinning at a fixed location.

Videos showing the stimulus conditions can be found in the Supplementary Material: spinning PLW (see Video 1; spinning rigid walking posture (see Video 2); an inverted PLW (see Video 3); an inverted rigid (see Video 4); a scrambled figure (see Video 5); A PLW walking along a circular path (see Video 6); and a rigid human in a walking posture traversing along a circular path (see Video 7).

## Methods

### Participants

Fifty-five observers (mean age 33, std 13, 29 females) participated in Experiment 1, and 31 participated in Experiment 2 (mean age 29, std 11, 18 females). All gave written informed consent and were compensated with a box of chocolate in Experiment 1, and two lottery tickets in Experiment 2. An additional sample of 20 participants was recruited for a control study in Experiment 2 (mean age 25.3, std 4.5, 16 females). They were informed that they could interrupt and leave the experiment at any time. No sensitive personal data was collected. Data were anonymized, and participants were neither physically nor psychologically manipulated.

### Stimuli

An algorithm developed by Poom (2023) generated movie sequences of a visually realistic human point-light-walker (PLW) where only dots on the major joints are visible. A previous version of this algorithm has been successfully used (Poom, 2012; Poom & Olsson, 2002). Colleagues and students have reported that the simulated point-light walker is very realistic.

The walker was specified by 13 dots located at the forehead, shoulders, elbows, hands, hips, knees, and heels. The limbs, shoulder, pelvis, and head motions were modeled with sinusoidally swinging hierarchically coupled pendulums simulating the motions in 3D space. The characteristic ballistic vertical body sway resulting from the motion of the feet as constrained by the ground is crucial for experiencing the walk as natural, otherwise, the limb motions resemble someone who treads water. The vertical body sway was produced by preventing the feet from traversing below the ground level while at least one foot (the heel or the forefeet) had ground contact throughout the walk cycle. In the simulation, pendulum motions of the feet were included to dampen the vertical bounce that otherwise appeared, but only dots specifying the positions of the heels were shown. The relative limb lengths and limb amplitudes during relaxed walk were obtained through measurements on real walking people. The projection surface was a vertical plane aligned with the lateral *x*-axis and vertical *y*-axis at *z* = 0 (which was the location of the spinning figure in Experiment 1, and the center of the circular path in Experiment 2). The projection point was located along the *z*-axis at 7,000 body lengths distance from *z* = 0. This approximates parallel projection to remove perspective cues and produce a perceptually ambiguously facing figure.

**Figure 2.**
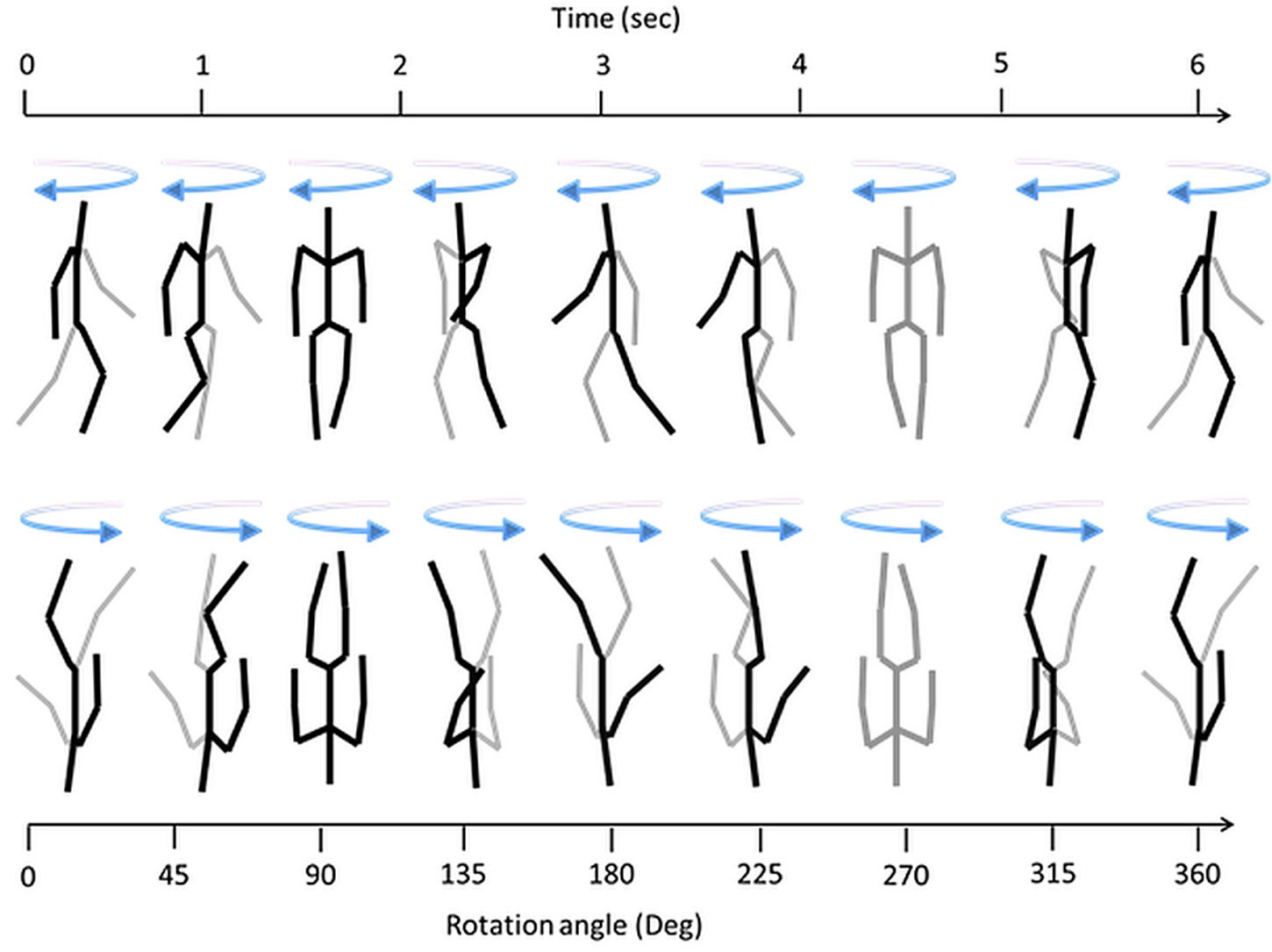
Stick-figures illustrating a complete 360° turn of the simulated walker during 6 s, from left to right. The black limb segments of the stick-figures are limbs simulated to be on the near side and the gray limb segments are on the far side of the figure. At 90° all segments are black to indicate that the simulated walker is facing straight toward the viewer. At 270°, all lines are gray to indicate that the walker is simulated facing straight away from the viewer. Perceived clockwise or anti-clockwise spinning is equally compatible with the motion presented on the screen. Inversion of the spinning figure reverses the simulated spinning direction which is clockwise for the upright and anti-clockwise for the inverted figure. The simulated spinning direction for the upright and inverted figures are implied by the arrows.

In Experiment 1, observers were presented with five movie sequences with spinning figures created by the PLW algorithm: walking upright, walking inverted, rigid upright (walking pose), rigid inverted (walking pose), and rigid scrambled. The rigidly spinning human figure in a walking pose was created by omitting the ambulating motions of the limbs. The rigid scrambled spinning figure was created by randomly scrambling the dots of the inverted rigid inverted figure along the vertical. Figure 2 shows nine sequential snapshots taken between 45° rotation steps for a rigidly upright and inverted human in a walking pose, illustrated with stick-figures. The inversion reversed the simulated spinning direction. Each of the five stimulus conditions with a spinning figure was shown in two sequentially presented blocks, each consisting of a 60-s movie that comprised 10 complete 360° turns. For each turn of the spinning motion, the walker completed five 1.2 s step-cycles composed of 40 movie frames, so a complete 360° turn was composed of 200 frames (1.8° rotation between frames). The spinning speed was 60°/s. A continuous walk while spinning, with no gaps in the walking phase at completed turns was produced by repeatedly presenting these 200 frames. In both experiments the height of the figure was 11 cm on the screen, corresponding to about 11° visual angle when viewed from a distance of 60 cm. For the spinning figures in Experiment 1 points were yellow (R, G, B values were 250, 250, 120) on a black background (0, 0, 0).

In Experiment 2, stimuli consisted of a walking human figure (see Supplementary Material Video 6) and a rigid human shape in a walking posture (Video 7) traversing a circular path seen from a side view. As for the spinning figures, these movies were produced by the PLW algorithm (Poom, 2023). Whereas the spinning figures in Experiment 1 were created by rotating the human figure about a vertical axis located at its spine, the circle walk was produced by displacing the human figure laterally ½ body length from the axis of rotation. Rotating this figure around the axis of rotation by 1.8° between each frame in the facing direction of the figure, produced the appearance of walk around the circle for the PLW and motion along the circle for the rigid human shape. The speed around the circle was matched with the walking speed implied by the step length and step frequency. The human figure was thus spinning 360° for each turn along the path. One lap around the circle took 6 s, so the spinning speed was identical to the spinning in Experiment 1 and the circular motion in Experiment 2.

Preliminary investigations using the figures moving in circular paths in Experiment 2 revealed that some participants were confused about the task of reporting reversals for the circular motion. This was due to the confound where the figure repeatedly changes direction of translation and facing direction on the screen when moving around in a circular path Therefore, the participants’ task was to report which side of the figure appeared closest every time it passed the midline of the screen. To distinguish the left and right sides of the figures the dots specifying the left hand, elbow, knee, and foot were colored pale blue (R, G, and B values were 153, 204, and 255), and corresponding dots on the right side were colored pale yellow (255, 255, 153). Hip, shoulder, and head dots were white (255, 255, 255) on a gray background (64, 64, 64). All other motion and size parameters were identical to the parameters used for the spinning figures in Experiment 1.

An additional control study was performed after Experiment 2 to ensure that the color differences between the spinning stimuli used in Experiment 1 and the circular motion used for the circular motion did not influence reversals. To this end, two movies of a spinning PLW were created: one with the same dot color as used for the spinning stimuli in Experiment 1, and the other with the same color difference as for the circular motion in Experiment 2. Both movies of a spinning PLW had the same gray background as in Experiment 2.

## Experiment 1

### Procedure and Analyses of Reversals From Spinning Motion

For each participant, the order between conditions was randomized but the same random order between stimuli was used in two blocks of 60 s each. So, the interval between two presentations of the same stimulus was similar (about 6–7 min). The initially perceived spinning direction was reported by pressing the F or K-key. The F-key (painted blue) was pressed when leftward spinning was perceived, i.e., clockwise as seen from above. The K-key (painted yellow) was pressed when rightward spinning was perceived, i.e., counterclockwise. Thereafter, the spacebar was pressed on occasions when the interpretation became mixed/uncertain. Reversals were reported by pressing the F-key when the spinning shifted from right or from mixed/uncertain direction to left (CW direction), and the K-key when the spinning shifted from left to right, or from uncertain direction to right (CCW direction).

The starting position of the spinning figures was 0° with the walker facing to the right of the screen. The walker was then spinning from a right-facing view to a left-facing view (at 180°) in a clockwise or counterclockwise spinning direction, equally compatible with the physical motion presented on the screen. If a clockwise left spinning was perceived between 0° and 180° then the walker adopted a facing pose. A change from clockwise to counterclockwise would thus mean that the walker was perceived to change from facing toward to facing away. Thus, for the upright figures, a left (CW) response made between 0° and 180° indicated that the figure reversed to facing toward the viewer (FTV+), whereas a right (CCW) response indicated that the figure faced away (FTV−). Between 180° and 360° a left (CW) response indicated FTV-, whereas a right (CCW) response indicated FTV+. In inverted conditions, this relation between angle and response type was reversed, since the simulated spinning direction was reversed as illustrated in Figure 2.

Traditional frequentist *p*-values (with an α-level of .05 if not otherwise stated) and, when possible, Bayes factors (BFs) were calculated by the freely available statistical JASP software (JASP Team, 2024). Unlike the *p*-value, the BF provides evidence both for and against the null hypothesis and BF_10_ is defined as the ratio between the likelihoods of the results given H_1_ and H_0_ (BF_01_ is the inverse ratio). All reported Bayes factors were computed using the default settings in JASP for the effect size priors. A flat prior to the alternative hypothesis, stating that there is a true correlation, was used for the correlation analyses. In Experiment 2, the Chi^2^ and a corresponding Bayesian joint multinomial contingency table test were applied (Jamil et al., 2017). As a guideline, it has been suggested that 1 < BF_10_ ≤ 3 is considered as anecdotal evidence, 3 < BF_10_ ≤ 10 is moderate evidence, 10 < BF_10_ ≤ 30 is strong evidence, 30 < BF_10_ ≤ 100 is very strong, and beyond that extremely strong evidence (Lee & Wagenmakers, 2014).

### Results for the Spinning Motion

**Figure 3.**
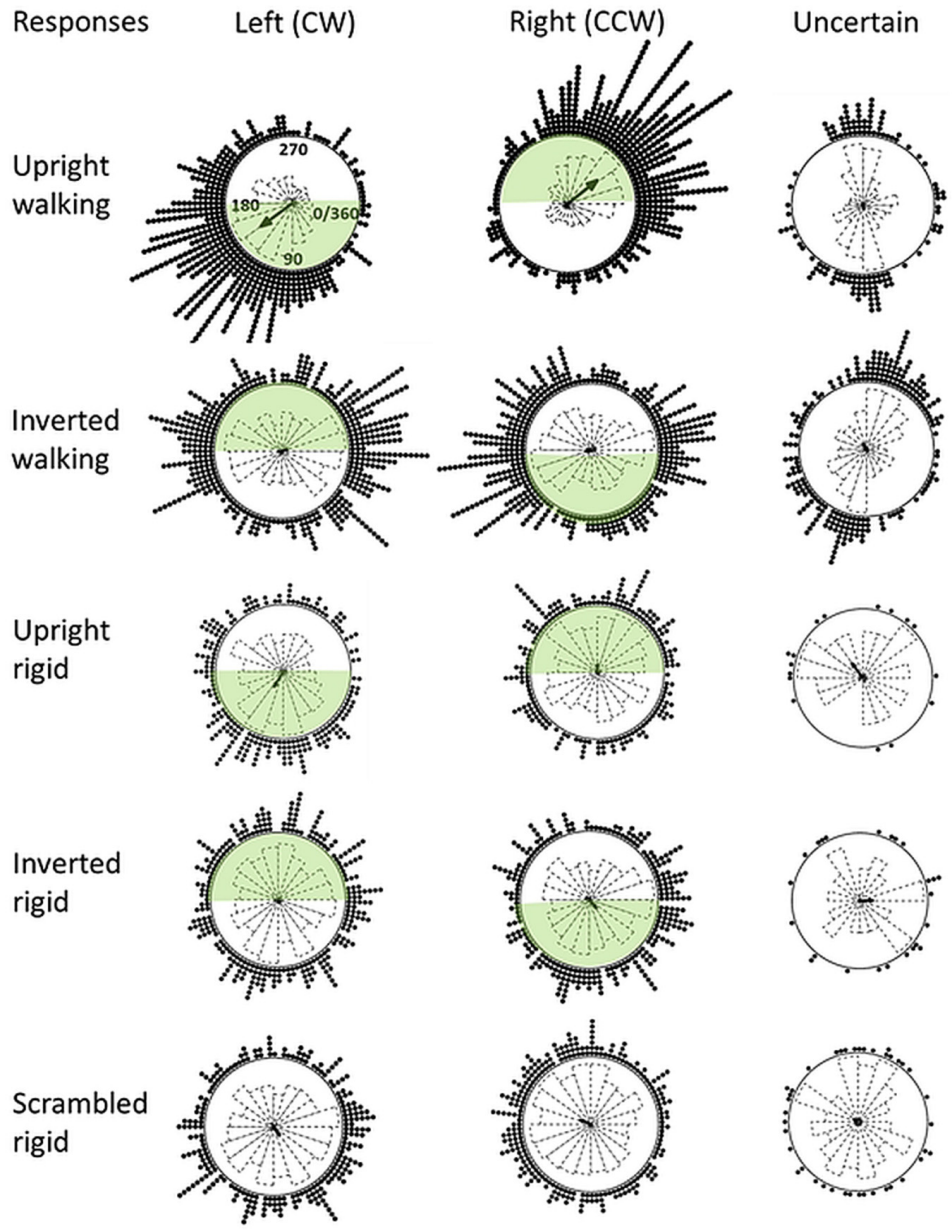
The circular plots display the timing of left (CW), right (CCW), and mixed/uncertain responses as a function of the angle of the spinning figures (angle is displayed in the top leftmost plot). The response distributions are visualized as stack-points and histograms. At the center of each circle is the main response vector. The walker is facing to the right at 0° (or 360°), and to the left at 180°. At 90°, 270°, and at oblique angles, the perceived facing direction is ambiguous and tied to the perceived spinning direction. Data are collapsed from all 55 participants during 2 min of observation comprising 20 turns in each condition. Responses within the shaded green area are reversals indicating that the figure changed to facing toward the observer (an FTV response), outside the green area are reversals indicating the opposite (a facing away response). Note that FTV responses are undefined in the scrambled condition.

The two blocks sequentially presented for participants were merged to result in 2 min of observation comprising 20 complete turns in each condition for each participant (no influence of blocks was obtained). Figure 3 shows the circular distribution plots, displaying the left (CW) right (CCW), and uncertain/mixed responses from the 55 participants, separately displayed for the 5 spinning figures. Responses are displayed as a function of the angle (0–360°) of the spinning figures. Each turn in a simulated clockwise direction took 6 s (the perceived direction was ambiguous). Zero degree indicate a side view where the human figure is facing to the right which is also displayed at the right in the circular distribution plots. Responses in the shaded green area indicate FTV responses (FTV+), whereas responses outside the green area indicate facing away responses (FTV−). Since the inversion reverses the simulated spinning direction as illustrated in Figure 2 the relation between the left and right responses and FTV+ and FTV− are reversed. No FTV responses could be defined for the scrambled figure since it had no specific facing side.

Inspection of Figure 3 shows that the left, right, and mixed/uncertain responses for the upright and inverted walker seem to depart from uniform distributions around the circle. For the upright walker the left and right responses were unimodal and located mainly within the green areas indicating a clear FTV bias. Whereas for the inverted walker, the distributions of the left and right responses appear bimodal with peaks at 0° and 180° with no FTV bias. Due to the response times, the responses were slightly delayed relative to the time when perceived reversals occurred. In a similar task, the delay was estimated to be about 500 milliseconds (Jackson et al., 2008). mixed/uncertain percepts mainly occurred for the spinning walkers, especially in the inverted condition when the figure was facing toward or facing away from the observer, just before the peak of reported left and right responses, indicating a transition phase between, or just before stable percepts. In the rigid conditions responses were few, and the response distributions in the scrambled rigid condition were similar to the upright and inverted rigid human shape conditions. So, the human shape did not seem to influence the perceived reversals in rigid conditions.

The frequentist Rayleigh uniformity test of circular data was applied to detect unimodal departures from uniformity in circular data (no corresponding Bayesian test was available). While Rayleigh's test of uniformity is recommended for circular unimodal data, it lacks statistical power in multimodal cases (Landler et al., 2018). Rao's spacing test is more powerful for multimodal distributions and small samples and was applied in cases where distributions in Figure 3 appear bimodal (if significant the Rao statistic is larger than the critical value). Across the five stimulus conditions, a Bonferroni corrected α-level = .005 was used for the analyses of the left and right responses which were treated as a distinct response category, whereas a Bonferroni corrected α-level = .01 was used for the mixed/uncertain responses which were treated as a separate response category.

The distributions of the left and right responses for the spinning walking figure in an upright position departed from uniformity (Rayleigh statistic = 582 and 554, both *p*'s < .001). These distributions are clearly unimodal as seen in Figure 3. For the inverted walker, the right response distributions departed from uniformity (Rayleigh statistic = .156, *p* < 001), whereas the left did not (Rayleigh statistic = .089, *p* = .02). From Figure 3 the distribution of these responses seems to be bimodal, and Rao's spacing test indicated a deviation from uniformity (Rao statistic = 173 > critical values 163 for α-level of .01, which was the minimum value provided by the statistics software). For none of the rigid conditions did the left and right responses depart from uniform distributions (.043 < Rayleigh statistics < .098, .15 < *p* < .61)

Figure 3 suggests a bimodal distribution for mixed/uncertain responses obtained in the upright and inverted walking conditions, but no significant departures from uniformity were obtained (Rayleigh statistic = .049 and .099, *p* = .738 and .020; Rao statistic values were 149 and 156, which are less than the critical value 166). The “mixed/uncertain” responses were few in all rigid conditions, and the Rao spacing test indicated no departures from uniform distributions (110 < Rao statistics < 125, which is less than the critical value 163).

In cases where departures from uniform distributions were found, the Watson-Wheeler test for circular data was applied to test if the two samples of left and right responses were differently distributed around the circle. The left and right response distributions were statistically different for the upright walker (*W* = 469, *p* < .001). No statistically significant difference between left and right response distributions for the inverted walker was obtained (*W* = 3.9, *p* = .14).

**Figure 4.**
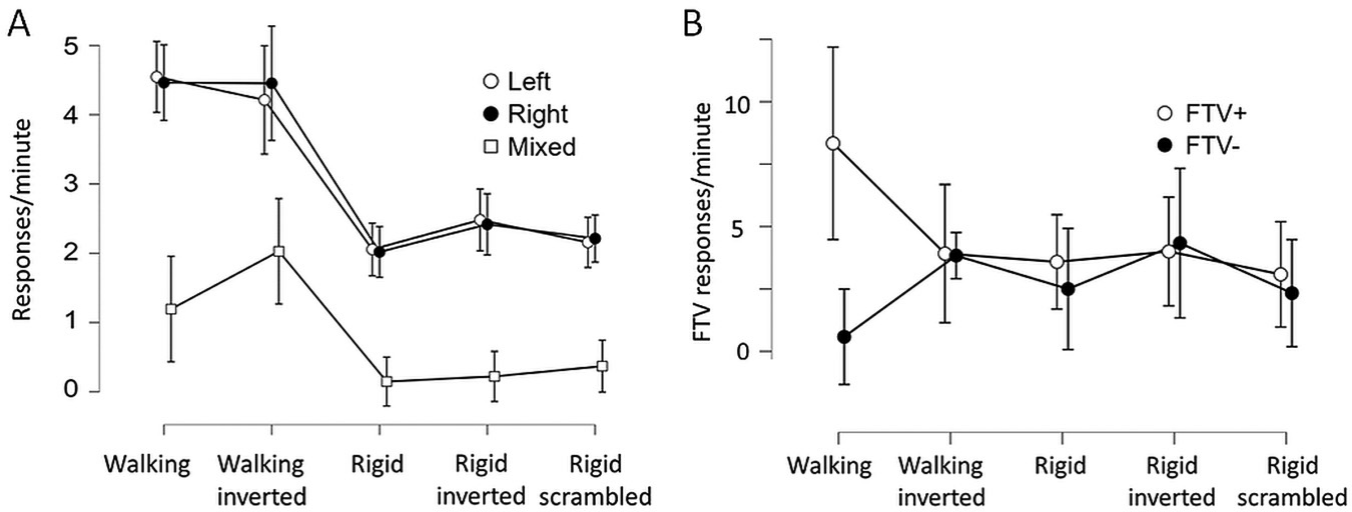
(A) The average number of perceived reversals to left and right spinning direction, and mixed/uncertain responses per minute during 1 min. (B) The left and right responses rearranged as the average number of facing the viewer and facing away from the viewer responses (during 1 min). For the scrambled condition, there is no FTV defined. The 95% CI calculated across the individual means is shown.

Figure 4A shows the frequency of left and right reversals per minute, and the mixed/uncertain responses. The reversal rate (combined left and right responses) differed between conditions as shown with a one-way frequentist and a Bayesian Repeated Measures ANOVA (F(4) = 20.5, *p* < .001; BF_10_ = 2.86 × 10^11^), as did the mixed response rate (F(4) = 7.95, *p* < .001; BF_10_ = 5920). Figure 4B shows the average number of facing the-viewer (FTV+) responses and responses indicating facing away from the viewer (FTV−) for each condition. An FTV bias was calculated as the difference between mean frequencies of the FTV+ and FTV− scores for each participant. A one-way ANOVA provided extremely strong evidence for this difference in FTV bias across conditions (F(4) = 11,5 *p* < .001; BF_10_ = 873 000). Sphericity corrections did not alter the results in cases when the assumption of sphericity was violated.

As a post hoc test for the aggregated left and right responses, the Bayesian two-tailed nonparametric paired sample Wilcoxon test was applied to test for differences since response frequencies were positively skewed. The Lee and Wagenmakers (2014) guidelines for interpreting Bayes factors was applied. The results provided extremely strong evidence for a difference between the walking and rigid conditions (990 < *W* < 1300, 652 < BF_10_ < 51 000), but no differences between the rigid stimulus conditions (329 < *W* < 616, .17 < BF_10_ < 1.3, ranging from moderate evidence for the absence of a difference to anecdotal evidence for a difference). The frequency of reversals experienced for the upright and inverted walkers was similar (*W* = 700, BF_10_ = .15, indicating moderate evidence for an absence of a difference).

Mixed/uncertain responses were more frequent for the inverted than for the upright walker (*W* = 177, BF_10_ = 7.7 considered as moderate evidence), and more frequent for the inverted walker than the rigidly spinning figures (704 < *W* < 780, 1153 < BF_10_ < 3300). The difference between the upright walker and the rigid figures indicates anecdotal to moderate evidence for a difference (177 < *W* < 254, 2.3 < BF_10_ < 5.7). No evidence for any difference between mixed/uncertain responses for the rigidly spinning figures was obtained (38 < *W* < 70, .23 < BF_10_ < 1.0).

An equal number of FTV+ and FTV− responses indicate no FTV bias (Figure 4B). For the upright walker, extremely strong evidence for a difference between FTV+ and FTV− was obtained (*W* = 1036, BF_10_ = 367). No reliable FTV bias was obtained in any other condition (470 < *W* < 731, .17 < BF_10_ < 1.87, ranging from moderate evidence for an absence of an FTV bias to anecdotal evidence for an FTV bias for the rigidly spinning figure). Thus, in contrast to Weech and Troje (2018) using inverted nonspinning PLW, no facing away bias for the inverted spinning PLW was observed.

Finally, nonparametric correlations between reversal rates were calculated since these data were skewed. Although not providing conclusive evidence, higher correlations indicate a correspondingly higher degree of involvement of common processes, or a common factor mediating the reversals. In Table 1, are the Kendall correlations between pairwise reversal rates observed in the different conditions in descending order. The correlations show that reversals were consistent across participants. In addition, reversal rates from the rigid SFM conditions were strongest related to each other, followed by correlations between the walking and rigid conditions. The weakest relations were found between the inverted biological motion and the other stimuli.

**Table 1.** Bayesian Kendall's tau correlations between pairwise reversal rates for the spinning figures (walking human, rigid human, and rigid scrambled figure).

		Kendall's tau B	BF_10_
Rigid inverted	Rigid scrambled	0.53 ***	973000
Rigid	Rigid scrambled	0.50***	199000
Rigid	Rigid inverted	0.49***	136000
Walking	Rigid scrambled	0.43***	6500
Walking	Rigid inverted	0.42***	2800
Walking	Rigid	0.41***	2500
Walking inverted	Rigid	0.30*	29
Walking inverted	Walking	0.30*	27
Walking inverted	Rigid inverted	0.24	4.8
Walking inverted	Rigid scrambled	0.20	1.8

* BF_10_ > 10, ** BF_10_ > 30, *** BF_10_ > 100.

## Experiment 2

### Procedure and Analyses of Reversals from the Circular Motion

For the circular motion, stimuli consisted of a PLW walking in circles, and a rigid human in a walking pose moving in circles while facing the motion direction. Observers were asked to report verbally which side of the figure appeared closest every time it passed the midline of the screen, while the experimenter noted the verbal reports. To distinguish the left and right sides of the figures, the dots specifying the left hand, elbow, knee, and foot were colored pale blue, and the corresponding dots on the right side were colored pale yellow. The hip, shoulder, and head dots were white on a gray background (see Stimuli section). At stimulus onset, the human figure started in a side perspective in the middle of the screen moving to the right. The direction around the circle could be perceived as clockwise or counterclockwise. An initial “Blue” report thus indicates that the figure started to move in a CW path, initially facing the observer, and if this motion continued so that the figure started facing away then “Yellow” would be reported next, etc. Thus, reporting which color appeared closer each time the walker passed the midline of the path was used as a proxy to perceived facing direction immediately before reaching this point.

During the 10 turns in the 1-min movie sequences the walker passed the midline 19 times which is the number of responses made by each observer in each condition. The initial response and the sequence of reversals were separately analyzed. An absence of any initial bias would result in an expected equal number of initial blue and yellow responses across the 31 participants. The sequence of the 18 responses after the initial response, was used to count the number of perceived reversals in each condition. An absence of reversals would result in repeated alternations between *Blue* and *Yellow* responses whereas perceived reversals resulted in the same color repeatedly reported. So, the number of reversals could be counted (see supplementary file for details). The frequentist Chi^2^ test and the Bayesian joint multinomial contingency table test on proportions were used for the statistical analyses. Preliminary investigations and a control study (see result section) showed that the color difference between the figures' left and right sides did not influence reversal rates.

Fifteen observers sat with the experimenter in a quiet room whereas the other 16 participated via Zoom while reporting color verbally to the experimenter (no difference in results was observed between these groups). The two stimulus conditions, walking and rigid, were presented in random order for each participant.

An additional sample of participants (*N* = 20) was recruited for a control experiment to investigate the impact of color on reversal rates for a spinning PLW as used in Experiment 1. The participants were instructed to count the reversals experienced during 1 min and report it verbally to the experimenter.

### Results for the Circular Motion

**Figure 5.**
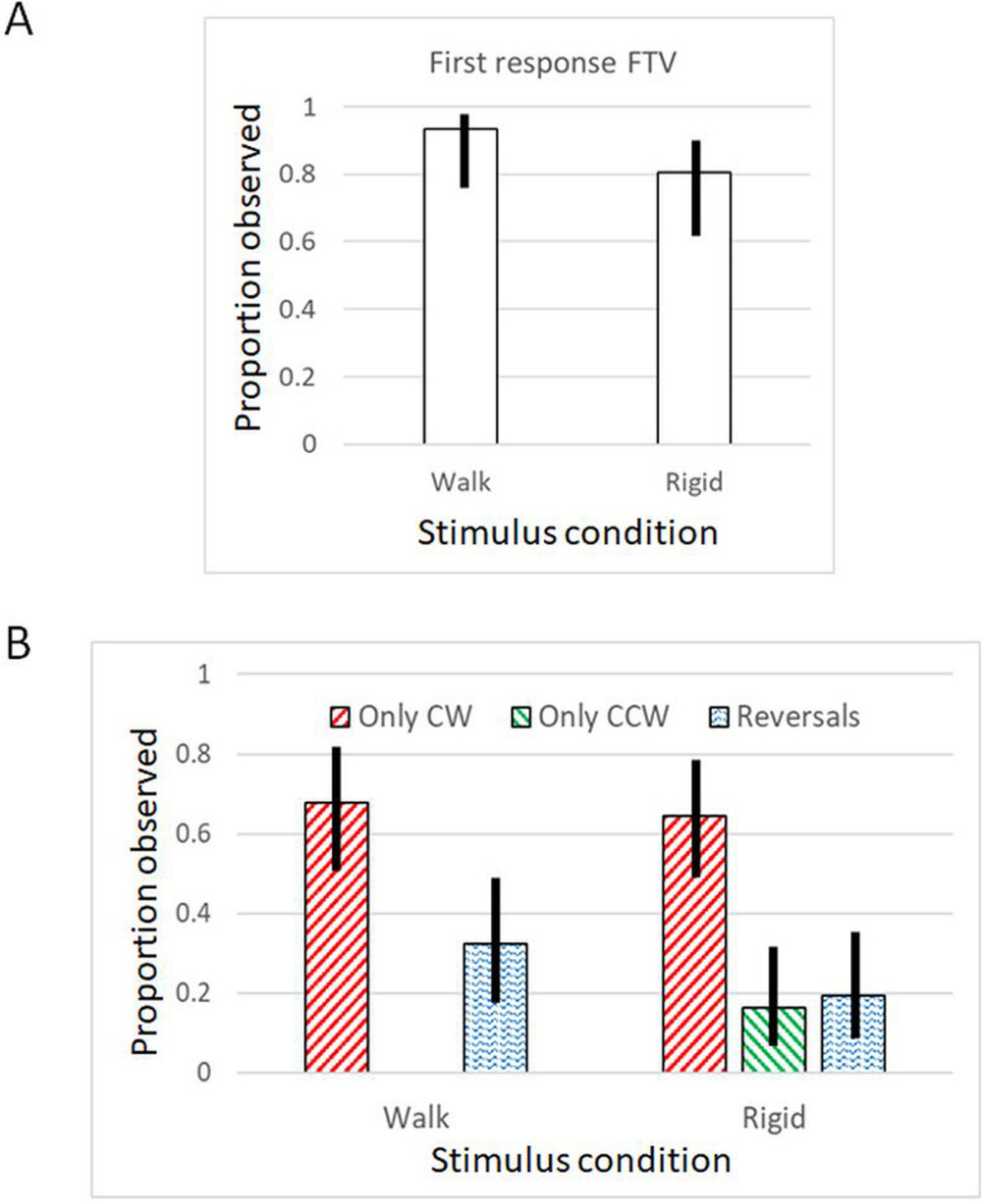
(A) Shown is the proportion of participants (out of 31) who initially in their first response reported perceiving the PLW moving in a circular path as facing the viewer (FTV) although the facing direction was ambiguous. The bars represent the two stimulus conditions: a walking and a rigid human shape in a walking posture while walking/traversing along its path. (B) The proportion of observers who experienced only clockwise or counterclockwise motion with no reversals during the whole observation, and the proportion of observers experiencing at least one reversal. Error bars show the 95% CI of proportions (the Wilson Score Interval).

Figure 5A shows the proportion of the 31 participants whose first response indicated a clear initial FTV response for the circular motion in walking and rigid conditions. This corresponds to an initially perceived CW motion. Figure 5B shows the proportion of observers who experienced only clockwise (CW) or counterclockwise (CCW) motions with no reversals. Also shown is the proportion of observers who experienced at least one reversal during the 1-min observations in the two stimulus conditions. After the initial response, a CW motion bias was apparent, and no observer experienced only CCW motion in the walking condition.

The proportion of first responses indicated a stronger FTV bias for the walker than for the rigid figure: Chi^2^ (1, *N* = 31) = 8.9, *p* = .003, and a Bayesian joint multinomial contingency table test found moderate evidence for this difference (BF_10_ = 5.3). The reversals were few. In the walking condition, on average, 1.5 reversals/min were reported: 7 observers made 38 FTV responses, whereas 3 observers made 8 facing away responses. In the rigid condition, on average, .52 reversals/min were reported: 4 observers made 1 FTV response each, whereas 1 observer made 12 facing away responses. The result is not a general CW motion bias since in a follow-up investigation where the PLW began moving to the left a corresponding CCW bias appeared. It is the initial FTV percept that determines the subsequently perceived stable direction.

To ensure that the color difference or background difference between the experiments did not influence reversals, an additional control study was performed. Twenty participants were presented with two movies of a spinning PLW: one with the same dot color as used in Experiment 1 and the other with the same color difference as in Experiment 2. Both movies had the same gray background as in Experiment 2. Participants were instructed to count reversals for 1 min and report it to the experimenter. On average 8.7 reversals/min were reported in the color condition, and 9.5 reversals/min in the no-color condition, which are similar to the 9 reversals/min obtained in Experiment 1 (the sum of left and right responses). A one-tailed nonparametric Wilcoxon-signed-rank frequentist and Bayesian tests found no reliable differences between reversal rates in the color and no color conditions used in the control experiment (*z* = 1.04, *p* = .16; *W* = 94, BF_10_ = .87). Reversal rates in the colored spinning control condition was 5.8 times faster than the 1.5 reversals/min for the similarly colored PLW walking in circles. A Mann–Whitney independent sample frequentist and Bayesian test was performed as a statistical test for this difference. The colored control condition resulted in reliably higher reversal rates than for the PLW walking in circles, although both had differently colored left and right sides (*U* = 470, *p* < .001; BF_10_ = 13). Thus, the color difference between the left and right sides of the PLW could likely not explain the difference in reversal rates found in Experiments 1 and 2.

In sum, for circular motion, there is a strong FTV bias at the first response that influences the subsequent perceived direction along the circle, which thereafter is reported as a stable clockwise motion with no reversals. Only a few observers made an initial facing-away response in the rigid condition which thereafter resulted in stable counterclockwise motion.

## Discussion

Reversals of the spinning stimuli were more than twice as frequent for the walking human (upright and inverted PLW) than for the rigid shapes (SFM). The reversal rate of a recognizable rigidly spinning human shape was no different from the reversals of the spinning rigid random structure. This suggests that the limb motions of the human shape trigger the reversals, not the human shape itself. Both the upright and inverted PLW resulted in nonuniform distributions of left (CW) and right (CCW) responses. But only the upright PLW resulted in an unimodal distribution of responses, with a clear FTV bias. For the inverted PLW, a bimodal response distribution was obtained with no FTV bias, which is contrary to the FTV bias found using an inverted nonspinning PLW (Weech & Troje, 2018). Few uniformly distributed reversals were obtained by the rigidly spinning shapes. This is contrary to Weech et al. (2014) who found an FTV bias for both walking and rigidly spinning stick-figures in a walking pose. Finally, the mixed/uncertain responses were rare for the rigidly spinning shapes. Mixed/uncertain responses were more frequent for the walking figures, especially for the inverted walker which was expected. Although an inspection of the circular distributions for the walking figures seems to indicate bimodal distributions of mixed/uncertain responses, the deviations from uniform distributions were not statistically significant.

The different results obtained from these studies may consist in that identification of a spinning PLW, especially when inverted, requires decomposition of the global rotation and local limb motions, which require the spread of attention over the stimulus. Stick-figures on the other hand may allow attention to be directed to local regions since the global organization is explicitly specified. Thus, the inverted nonspinning PLW may enable attention to be directed to the lower part of the stimulus to produce a convexity bias at the elbows, whereas the inverted spinning PLW requires attention spread over the whole stimulus to enable identification. In addition, reversal rates for the inverted walker were the least correlated with the other conditions, which could result from stronger recruitment of top-down processes to assist in the identification process. Strong correlations were obtained between the reversal rates for the rigidly spinning shapes (combined left and right responses). Correlations between reversals for the upright walker and the rigid conditions were also found, although contrary to the rigidly spinning shapes the upright walker produced a nonuniform distribution of responses and an FTV bias. High correlations could result from the involvement of common processes mediating the reversals.

The task of reporting reversals where the whole figure is perceived as spinning in the same direction and reverses coherently must rely on a global organization from motion. It is rarely, or never, the case that the upper and the lower body spin in opposite directions, or that independent reversals occur for other components. For a rigidly spinning SFM stimulus, the opposite motion directions of the dots specify the near and far sides of the structure, although ambiguously. For a spinning PLW on the other hand, identification requires decomposition of the common rotation and the periodic motion of the limbs. Mixed spinning directions can occasionally be perceived for the inverted PLW, as spontaneously reported during informal demonstrations. This is likely triggered by the motions of the limbs back and forth since the rigidly spinning inverted human figure is perceived to spin coherently. Identification is facilitated by top-down processes, for example by informing observers about the nature of the motion display which likely activates reverberations of bottom-up and top-down processes (Pastukhov, 2017). The much higher reversal rates for the spinning PLW compared to the rigidly spinning shapes can be accounted for by an attractor network model where reversals are induced by noise (Moreno-Bote et al., 2007). The higher rates of mixed/uncertain responses for the walking stimuli compared to the rigidly spinning shapes suggests that limb motions introduce such noise. In addition, reversals for the walkers occur at specific angles which is also the case for some rigidly spinning nonuniform SFM shapes (Brouwer & van Ee, 2006; Pastukhov et al., 2012; Wallach & O’Connell, 1953). The nonuniform shape of a PLW has been proposed to account for the rapid reversals previously observed for a spinning PLW (Jackson et al., 2008). Although all stimuli used here were nonuniform, reversals were uniformly distributed for the rigidly spinning stimuli, and the reversal rate was much slower than for the PLW, which supports the attractor model where limb motions introduce noise.

In Experiment 2, the walking and rigid human figures moved along a circular trajectory facing the motion direction. The results revealed a strong initial FTV bias for both the walking and rigid human in a walking pose traversing a circular path. As the motion continued few reversals were reported and the PLW was perceived to move steadily along the circular path in the same direction as initially reported. The difference in reversal rates for spinning and circular motion is consistent with an experience-based influence. Prior experiences of other walking humans spinning at a fixed spot are rare compared to other people walking in circular paths. This makes the interpretation of the ambiguously spinning PLW more prone to reverse to keep facing and approaching the viewer (an FTV bias). The circular walk resolves the perceptual ambiguity of the PLW, similar to how a view from above helps to resolve the perceptual ambiguity of a nonspinning PLW (Zhang et al., 2017).

Previous experiences influence perception and may lead to various interacting biases. An up-down asymmetry caused by gravity and a ground plane has formed our visual experiences and led to various classical size and brightness illusions (Poom, 2020). Also, as noted by Johansson (1973) an inverted point-light biological motion display is much more difficult to recognize than an upright display. Similarly, inverted stills of human body positions are much more difficult to recognize than other types of nonanimate objects (Reed et al., 2003), as are recognition of inverted faces (Yin, 1969). When viewers’ perspective can be interpreted as a view from above or from below, the view from above interpretation is preferred (Zhang et al., 2017). Interestingly, directly after treatment for congenital blindness a human PLW can be recognized in both upright and inverted conditions, whereas animal PLW cannot be recognized (Ben-Ami et al., 2022). This suggests that the inversion effect of human motion patterns depends on later visual experience, whereas a visual representation may form from much earlier experiences of proprioceptive information about one's own limb movements not specified in relation to the up and down directions (Ben-Ami et al., 2022). Accordingly, this could explain infants perceptual sensitivity to biological motion (Falck-Ytter et al., 2011; Fox & McDaniel, 1982; Simion et al., 2008). In addition, prolonged microgravity during space flight, and time spent in a 6° head-down-tilted bed rest, reduces the inversion effect in biological motion perception, but not for inverted faces, suggesting that humans adaptively utilize gravity as an embodied constraint to facilitate the perception of life motion signals (Wang et al., 2022). This reduced inversion effect was also accompanied by strengthened connectivity between cortical areas dedicated to biological motion and vestibular gravity estimation.

The FTV bias may result from attentional preferences. Brain imaging studies suggest that approaching individuals attract our attention more than receding individuals (Pelphrey et al., 2004; Saygin et al., 2004). This is likely because approaching people is more important to keep track of since it may result in social interactions and even hostile confrontations. This could then result in a bias to interpret an ambiguous PLW as approaching. This explanation is similar to the explanation where the FTV bias results from avoiding costly mistakes, but the origin of the bias is different. In the former explanation, the FTV bias is due to prior perceptual experiences whereas the latter explanation assigns the cause to a FTV bias to a social interpretation of the stimulus. A prediction from the experience-based hypothesis is that people attending other people facing away for an extended period would subsequently experience less FTV bias or even a facing away bias, similar to a priming effect. For example, in crowded environments people attend to other people facing away (Berton et al., 2022), likely to avoid colliding with these people. People exposed to other people facing away for a prolonged period, by, e.g., being exposed to crowded environments, could be used to test this hypothesis of an experience-based influence of the FTV bias in a future study. A related example is the reduction of the inversion effect by reduced gravity (Wang et al., 2022). It is also known that long-duration spaceflight decreases the view-from-above bias and therefore increases the depth ambiguities of reversible wire-frame figures like the Necker cube (Clément et al., 2015). The experience-based FTV might also be tested experimentally by priming. For example, observing a walker facing away should cause a subsequently observed ambiguous PLW to be perceived as facing away as well. Although priming with short exposure can efficiently influence the perception of ambiguous SFM stimuli (Poom & Matin, 2022), it is uncertain if short-exposure priming influences the perception of an ambiguous PLW.

Differences in the processes of perceiving biological motion and rigid SFM have been investigated before. For example, when embedded in noise, the direction of a PLW is integrated over time and increases with increasing gait period but is fixed for common unidirectional planar motion (Neri et al., 1998), and a rigid human point light shape in a walking posture (Poom & Olsson, 2002). Still, the task of reporting direction can be accomplished by local cues (Troje & Chang, 2023). Different processes are recruited for biological point-light motion displays depending on the task. Short-term visual memory precision of the walking direction of a PLW (which is a local process) does not decay during 10 s of retention, whereas short-term memory of gender-stereotypical gait patterns does decay (which require global processes, i.e., comparison of hip vs. shoulder sway) (Poom, 2012). Such dissociation may involve separate contributions of active and passive processing of motion stimuli such as global and local information. For example, biological motion point-light displays seem to activate “life detectors” that signal the motion direction from the local motion of the feet (Troje & Chang, 2023; Troje & Westhoff, 2006). Shiffrar et al. (1997) in targeting global processes showed that observers cannot recognize moving rigid objects through apertures, although they can easily recognize upright walking stick-figures viewed through the same apertures. Rigid SFM also seems to involve multiple processes. For example, no correlation is found between reversals from an SFM object involved in spinning motion and oscillating wobbling motion. Spinning SFM results in more frequent reversals than if the motion oscillates back and forth, suggesting that oscillations hamper the adaptation-recovery cycles involved in the causation of perceived reversals (Poom, 2000; 2024). These results suggest that both low-level and higher-level processes contribute to reversals, such as switching between hypotheses to explain the sensory signals (Poom, 2024), which are likely influenced by past occurrences.

Since biological motion perception was first demonstrated by Johansson (1973), researchers have explored boundary conditions for biological motion perception (i.e., Ahlström et al., 1997; Johansson, 1976; Neri et al., 1998; Poom & Olsson, 2002). Biological motion stimuli have since then been used for other purposes than investigating the mechanisms for perceptual organization (Troje, 2013). Biological motion conveys information about affective states, intentions, and other higher-order information (Runeson & Frykholm, 1983). Research using biological motion stimuli has made significant contributions to visual and social cognition (for reviews see Blake & Shiffrar, 2007; Pavlova, 2012; Steel et al., 2014), and to clinical psychology (Henry et al., 2012; Kim et al., 2005; Okruszek et al., 2018; Okruszek & Pilecka, 2017; Pavlova, 2012; Tian et al., 2023; Todorova et al., 2019). In addition, biological motion perception from point-light displays is prevalent across a wide range of species, including mammals, fish, and even spiders (Blake, 1993; De Agrò et al., 2021; Ishikawa et al., 2018; MacKinnon et al., 2010; Schluessel et al., 2015; Troje & Aust, 2013). For humans, biological motion conveys information not only about distal configuration but also about action, intention, and emotion and therefore involves multiple brain areas that influence the interpretation of ambiguous biological stimuli (Grossman et al., 2000; Saygin et al., 2004). Here, it is shown that the FTV bias from a spinning PLW is crucially dependent on the limb movements of an identifiable human figure. An identical spinning rigid human in a walking posture does not result in any FTV bias. As described, some of the results presented here using spinning point-light displays are in conflict with previous studies using spinning stick-figures (Weech et al., 2014), or a nonspinning PLW (Weech & Troje, 2018) indicating that such differences influence perception, possibly due to different spread and focus of attention. Also, when the PLW is spinning at a fixed location it is less stable and reverses more frequently than if the spinning motion is caused by traversing a circular path, and likely also less stable than a nonspinning walker that rarely reverses. Thus, multiple factors contribute to the perception of an ambiguous PLW. When information is minimized, then prior experiences, serving adaptive functions in visual perception, cause various biases that are integrated to resolve perceptual ambiguities.

## Supplemental Material

**Figure:** Video 1.

**Figure:** Video 2.

**Figure:** Video 3.

**Figure:** Video 4.

**Figure:** Video 5.

**Figure:** Video 6.

**Figure:** Video 7.

sj-docx-1-ipe-10.1177_20416695251342410 - Supplemental material for Dynamics of visual reversals from ambiguous spinning biological-motion and rigid structure-from-motion Supplemental material, sj-docx-1-ipe-10.1177_20416695251342410 for Dynamics of visual reversals from ambiguous spinning biological-motion and rigid structure-from-motion by Leo Poom in i-Perception
