# Supplementary material for: Dynamics of visual reversals from ambiguous spinning biological-motion and rigid structure-from-motion
Source: Iperception. 2025 May 21;16(3):20416695251342410. doi: 10.1177/20416695251342410 (PMC12120536; doi:10.1177/20416695251342410)
Supplement: sj-docx-1-ipe-10.1177_20416695251342410 - Supplemental material for Dynamics of visual reversals from ambiguous spinning biological-motion and rigid structure-from-motion [file sj-docx-1-ipe-10.1177_20416695251342410.docx]

**Supplementary methods**

Figure 8 A is a schematic top-view of the circle walk, with the observer on the right, and the footsteps of the PLW along the path on the left. Blue and yellow footsteps indicate the walkers left and right side. At stimulus onset the figure started in a side perspective moving to the right, the direction around the circle could be perceived as clockwise or counterclockwise. The rectangle located at the mid position of the circular path, as seen from the observer, indicates the position of the PLW when observers responded which color of the PLW was perceived nearest. The PLW was then shown in a lateral view moving to the right or to the left, but the direction along the path, clockwise or counter-clockwise, was ambiguous. Observers verbally reported the color of the dots that appeared closest each time the PLW passed the midline of its path (when the figure was perceived from a side view) to the experimenter who noted each response: “Blue” responses when the left side of the PLW and “Yellow” responses when the right side of the PLW was oriented toward the observer (perceived closest). Since a clockwise direction around the circle is simulated and the PLW is marked with blue dots on its simulated left side, “Blue” responses mean that the PLW was facing the viewer and “Yellow” responses were given when the PLW was perceived facing away immediately before passing the midline. In short, blue and yellow responses inform about the perceived direction along the path, clockwise or counter-clockwise. “Blue” responses indicate perceived FTV whereas “Yellow” responses indicate facing away (before reaching the midline of the path).


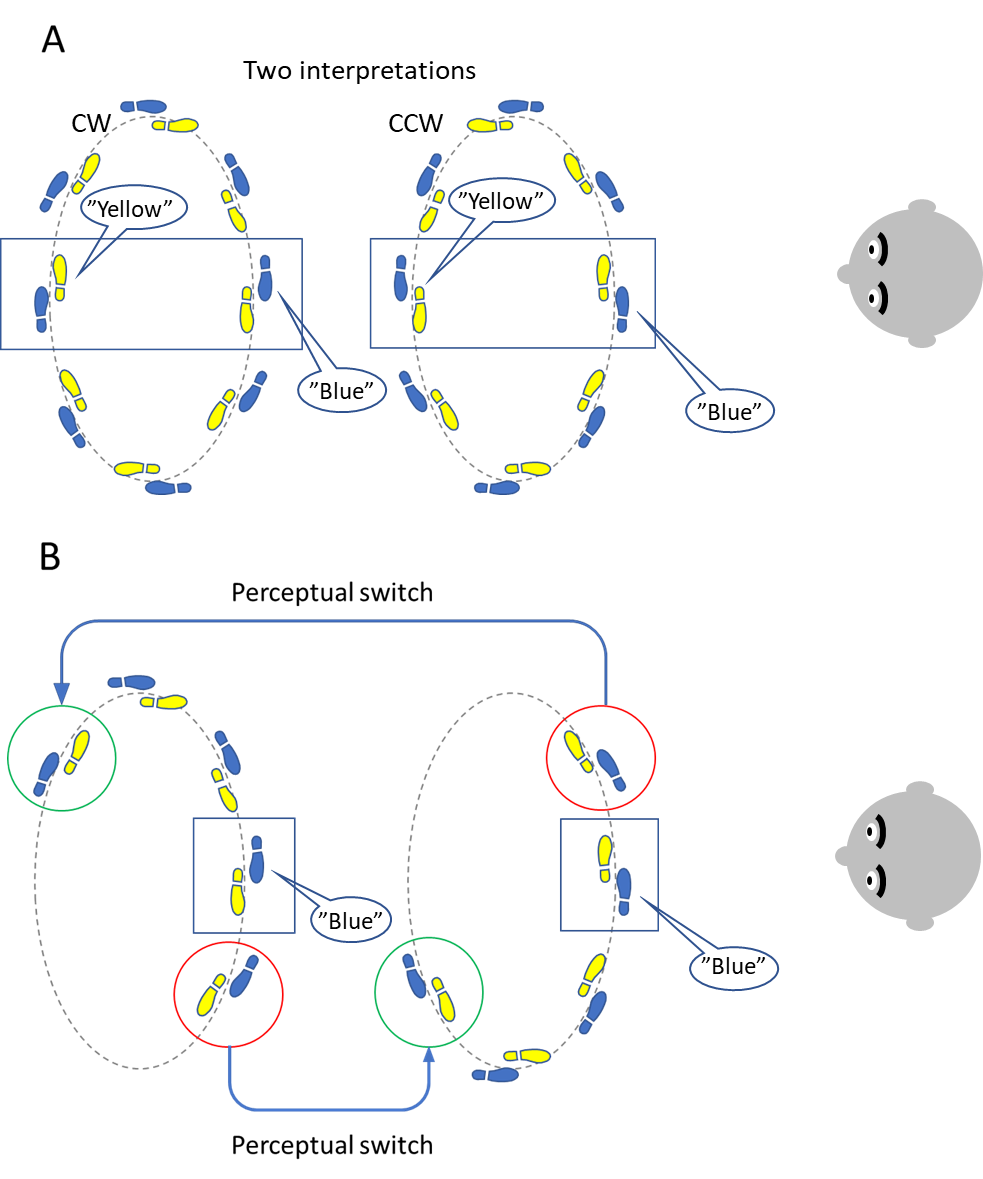


Figure 8. (A) A schematic illustration with a view from above of the footsteps of the perceived direction of walk along the circular path (vertically elongated in the illustration). The perceived direction of walk is ambiguous and can be perceived as clockwise (CW) or counter clockwise walk (CCW). Within the rectangle are the walker positions at the time of response (a side view of the walker), “Blue” or “Yellow” perceived as nearest. At stimulus onset the figure started in a side perspective moving to the right. (B) A facing the viewer bias results in reversals when walker begin to face away from the observer as indicated by the footsteps encircled in red, the alternate “facing toward” percept is encircled in green.

During perceived continues circular motion along the path the PLW alternates between facing toward and facing away and the responses should also alternate between “Blue” and “Yellow” (Figure 8A). An initial “Blue” response means that the PLW at its first turn was perceived as facing the viewer moving in a clockwise direction, and an initial “Yellow” response means that it was perceived as facing away from the viewer moving in a counterclockwise direction.

A reversal to facing the viewer may occur when the walker begins facing away as shown by the arrows from the red-encircled footsteps pointing to the green encircled footsteps in Figure 8B. This will result in two consecutive “Blue” responses (a FTV+ response). Similarly, a reversal from facing the viewer to facing-away results in two consecutive “Yellow” responses (FTV-).
